# Single-cell RNA and T-cell receptor sequencing unveil mycosis fungoides heterogeneity and a possible gene signature

**DOI:** 10.3389/fonc.2024.1408614

**Published:** 2024-08-07

**Authors:** Nalini Srinivas, Lukas Peiffer, Kai Horny, Kuan Cheok Lei, Terkild B. Buus, Linda Kubat, Meng Luo, Menghong Yin, Ivelina Spassova, Antje Sucker, Farnoush Farahpour, Jan Kehrmann, Selma Ugurel, Elisabeth Livingstone, Thilo Gambichler, Niels Ødum, Jürgen C. Becker

**Affiliations:** ^1^ Translational Skin Cancer Research, German Cancer Consortium (DKTK), University Medicine Essen, Essen, Germany; ^2^ German Cancer Research Center (DKFZ), Heidelberg, Germany; ^3^ Department of Dermatology, University Hospital Essen, and German Cancer Consortium (DKTK) partner site Essen/Düsseldorf, Essen, Germany; ^4^ LEO Foundation Skin Immunology Research Center, Department of Immunology and Microbiology, University of Copenhagen, Copenhagen, Denmark; ^5^ Bioinformatics and Computational Biophysics, University Duisburg-Essen, and Group of Molecular Cell Biology, Institute for Cell Biology (Cancer Research), University Hospital Essen, Essen, Germany; ^6^ Institute of Medical Microbiology, University Hospital Essen, University of Duisburg-Essen, Essen, Germany; ^7^ Department of Dermatology, Ruhr-University Bochum, Bochum, Germany; ^8^ Department of Dermatology, Dortmund Hospital, University Witten/Herdecke, Dortmund, Germany

**Keywords:** CTCL, heterogeneity, malignant T cells, single-cell RNA sequencing, TCR sequencing, gene signature

## Abstract

**Background:**

Mycosis fungoides (MF) is the most common subtype of cutaneous T-cell lymphoma (CTCL). Comprehensive analysis of MF cells *in situ* and *ex vivo* is complicated by the fact that is challenging to distinguish malignant from reactive T cells with certainty.

**Methods:**

To overcome this limitation, we performed combined single-cell RNA (scRNAseq) and T-cell receptor TCR sequencing (scTCRseq) of skin lesions of cutaneous MF lesions from 12 patients. A sufficient quantity of living T cells was obtained from 9 patients, but 2 had to be excluded due to unclear diagnoses (coexisting CLL or revision to a fixed toxic drug eruption).

**Results:**

From the remaining patients we established single-cell mRNA expression profiles and the corresponding TCR repertoire of 18,630 T cells. TCR clonality unequivocally identified 13,592 malignant T cells. Reactive T cells of all patients clustered together, while malignant cells of each patient formed a unique cluster expressing genes typical of naive/memory, such as *CD27, CCR7* and *IL7R*, or cytotoxic T cells, e.g., *GZMA, NKG7* and *GNLY*. Genes encoding classic CTCL markers were not detected in all clusters, consistent with the fact that mRNA expression does not correlate linearly with protein expression. Nevertheless, we successfully pinpointed distinctive gene signatures differentiating reactive malignant from malignant T cells: keratins (*KRT81*, *KRT86*), galectins (*LGALS1*, *LGALS3*) and S100 genes (*S100A4*, *S100A6*) being overexpressed in malignant cells.

**Conclusions:**

Combined scRNAseq and scTCRseq not only allows unambiguous identification of MF cells, but also revealed marked heterogeneity between and within patients with unexpected functional phenotypes. While the correlation between mRNA and protein abundance was limited with respect to established MF markers, we were able to identify a single-cell gene expression signature that distinguishes malignant from reactive T cells.

## 1 Introduction

Cutaneous T-cell lymphomas (CTCLs) comprise a heterogeneous group of non-Hodgkin lymphomas that – with the exception of Sézary syndrome (SS) – characterized by localization of neoplastic T lymphocytes to the skin, with no evidence of extracutaneous disease at the time of diagnosis (1). Mycosis fungoides (MF) represents the most common type, accounting for approximately 70% of all CTCL. It presents with patches and plaques and tends to progress over years or even decades to more infiltrated plaques and tumors. MF comprises several subtypes with different characteristics and clinical courses and has an indolent course in most patients. However, 5 to 10% of patients develop aggressive disease associated with high mortality (2). It is generally acknowledged that MF progression is accompanied by a shift from a Th1 toward a Th2 polarization of the reactive infiltrate in the cutaneous lesions. Still, neither the exact mechanisms of disease progression nor valid prognostic markers are well established since it is notoriously difficult to discriminate malignant from reactive T cells. Recent advancements in technology, enabling the concurrent sequencing of T-cell receptors (TCR) and RNA within individual cells, greatly simplify the process of making this differentiation (3). The first single-cell (sc) transcriptomic analysis for CTCL was performed on peripheral blood of patients with SS demonstrating heterogeneity of malignant CTCL cells (4). Similar analyses of 4 MF patients confirmed distinct gene expression patterns for malignant T cells (5); however, this study did not identify the TCR of the MF cells. Recent studies demonstrated the benefits of combined scRNA and scTCRseq that showed high degree of inter and intra-patient heterogeneity with distinct transcriptomic profiles within malignant T-cell population in SS (4, 6, 7). In another study, Herrera et al., identified the same malignant T-cell clone in blood and skin in 4 SS and 1 MF patient (8). Interestingly, the skin microenvironment promoted an activated and proliferative state of the malignant T-cell clone. Similarly, in a case report, an individual MF cell clone could be followed in different tissue compartments, namely skin, lymph node and blood (9). The malignant T-cell clone exhibited a gene expression pattern similar to tissue-resident memory T cells when present in the skin, whereas in the lymph node and blood, the gene expression was more similar to a central memory T cell.

Here, we expanded these investigations by analyzing skin biopsies of MF lesions from 12 patients. In 9 of these, we isolated ample viable T cells - but 2 had to be excluded for other reasons - enabling us to conduct combined scRNA and scTCRseq. Remarkably, in all 7 instances, we unequivocally pinpointed the malignant T-cell clones. By merging the power of both methods, we unveiled a captivating dual revelation: not only does it validate the remarkable diversity within mycosis fungoides cells across and within individuals, but it also unearths a hitherto undescribed CD4+ cytotoxic subtype, adding an intriguing layer to our understanding.

## 2 Materials and methods

### 2.1 Patients

From specimen excisions taken for confirmation of the diagnosis or for follow-up of stage ≥IIA MF at the dermatology departments of the University Hospital Essen and the Ruhr-University Bochum, part of the tissue from 12 patients could also be used fresh for further scientific investigations. The ethics committee of the University Duisburg-Essen approved the project (18–8230-BO), which was conducted in accordance with the Declaration of Helsinki. Written informed consent from each patient was obtained prior to any measures.

### 2.2 Single-cell RNA and TCR sequencing

Native tissue of cutaneous MF lesions was immediately (i.e., within 2 hours after excision) transferred to single-cell suspension. Briefly, tissue cubes of 2 mm edge length were minced in gentleMACS (Miltenyi Biotec, Bergisch Gladbach, Germany) using program “h_tumor_01”, followed by program “h_tumor_02” twice with 30 min intervals in an enzyme mix consisting of 200 µl Enzyme H, 100 µl Enzyme R and 25 µl Enzyme A (catalogue #130–095-929 Miltenyi Biotec). After three washes with PBS/0.05% bovine serum albumin (BSA), cells were passed through a 100 µm cell strainer. The number and viability of T cells in the single-cell suspension was assessed by flow cytometry using antibodies against CD3 (catalogue #1P-514-T025, EXBIO Praha, Vestec, Czech Republic) and CD45 (catalogue #1A-222-T100, EXBIO Praha) together with 7-aminoactinomycin (7AAD, catalogue # SML1633–1ML, Merck, Darmstadt, Germany) in a CytoFLEX (Beckman Coulter, Brea, CA, USA). More than 80% living cells containing at least 30% T cells were required for scRNA and scTCRseq using the Single Cell 5’ Library & Gel Bead Kit v1.1 (catalogue #1000165, 10xGenomics, Pleasanton, CA, USA) for 12,000 cells at a concentration of 600 cells/µl. After Gel Bead-In EMulsions (GEM) generation, the mRNA was reversely transcribed and cDNA amplified for library construction. The gene expression libraries were sequenced at the DKFZ Genomics and Proteomics core facility on a NovaSeq 6000, paired-end with 26 cycles of the forward and 74 cycles on the reverse read, the TCR libraries on a NextSeq 550 platform with Paired-End 150bp Mid-Output. Samples were sequenced at an average of 64,251 reads per cell. The number of recovered cells varied between 1,092 and 9,846 cells with an average of 5,246 cells (43,7% recovery) and 1,480 identified genes per cell.

### 2.3 Data analysis

Sequencing data was processed with the Cell Ranger pipeline (v3.0, 10xGenomics) with default settings respective for gene expression using the hg19 reference genome; TCR libraries were handled accordingly. Quality metrices for filtering living cells were as follows: percentage of mitochondrial gene expression <12%, unique molecular identifier (UMI) count >500, gene count >200 and number of expressed housekeeping genes >40. Number of expressed housekeeping genes was determined by housekeepers identified from Tirosh et al. with at least one UMI count (10). For the subsequent analysis, the Seurat R package (v3.2.2) was used (11). Additional analyses were performed in R (v3.40) using the indicated packages. Samples were individually processed. After log-normalization of gene-expression counts cell cycle effects were regressed using S- and G2M scores depending on markers from Tirosh et al. as well as mitochondrial gene expression (10, 12). The 2000 most variable genes were selected and principal components (PC) were calculated. Between 15 and 25 PCs were used for downstream analysis determined by the inflection point in an elbow plot ranking the PCs by their variance. Cell clusters were determined using shared nearest neighbor modularity optimization using the Louvain algorithm (at resolution of 0.5) and visualized in reduced dimensionality using uniform manifold approximation and projection (UMAP). Significantly differentially expressed genes were assessed using Wilcoxon rank sum test (ln fold change (FC) > |0.25|, Bonferroni-adjusted P value < 0.05). Finally, clonotype information was added to the meta data of the respective Seurat object based on the “filtered_contig_annotations” file. Of note, the identified malignant T-cell clone in some patients was split into different clusters based on the detection of either only α, only β or α and β chains, which were brought together again for subsequent analyses. After processing each sample individually, they were merged with the “merge” command of Seurat and subsequently log-normalized again.

Large-scale chromosomal copy number variations were calculated with the inferCNV R package of the Trinity CTAT project ("https://github.com/broadinstitute/inferCNV") and depicted as heatmap. Healthy T cells were used as reference.

To determine RNA velocities, we used the R package velocyto (13). Velocyto aims to predict future transcriptional cell states by assuming that unspliced, intronic reads can be interpreted as nascent RNAs. The ratio of unspliced and spliced reads can be used to infer RNA turnover rates (aka RNA velocities) and for estimation of future transcriptome states. These futures states of a cell can also be projected on lower dimensional embeddings to visualize transition of cell clusters. Here, transitions are visualized as arrows. Arrow origins indicate the current and arrow tips the estimated future transcriptional state. We applied the velocyto package following the R tutorial according to La Manno et al. using default settings (13). In order to quantify transition distances, we used principal component analysis (PCA) embedding. In the PCA space, arrow lengths scale linearly with the dimension and can then be associated with changes in gene expression; e.g. long arrows indicating large changes. Thus, populations with short arrows indicate stationary cells while populations with longer arrows transitioning cells. To describe transition dynamics within and across samples, we measured the transition distance (i.e., the length of the arrow) from current to future cell states in the PCA reduced representation. Transition distances were calculated by: td = (ΔPC1^2^ + tΔPC2^2^)^1/2^ for each cell and normalized to a value between 0 and 1 (td_normalized = (td - min(td))/max(td)). Median transition distances and the inter-quartile range (IQR: calculated as 75% quantile - 25% quantile) were used to compare dynamics among samples and variations within a sample.

## 3 Results

### 3.1 Patients’ characteristics

**Figure 1:**
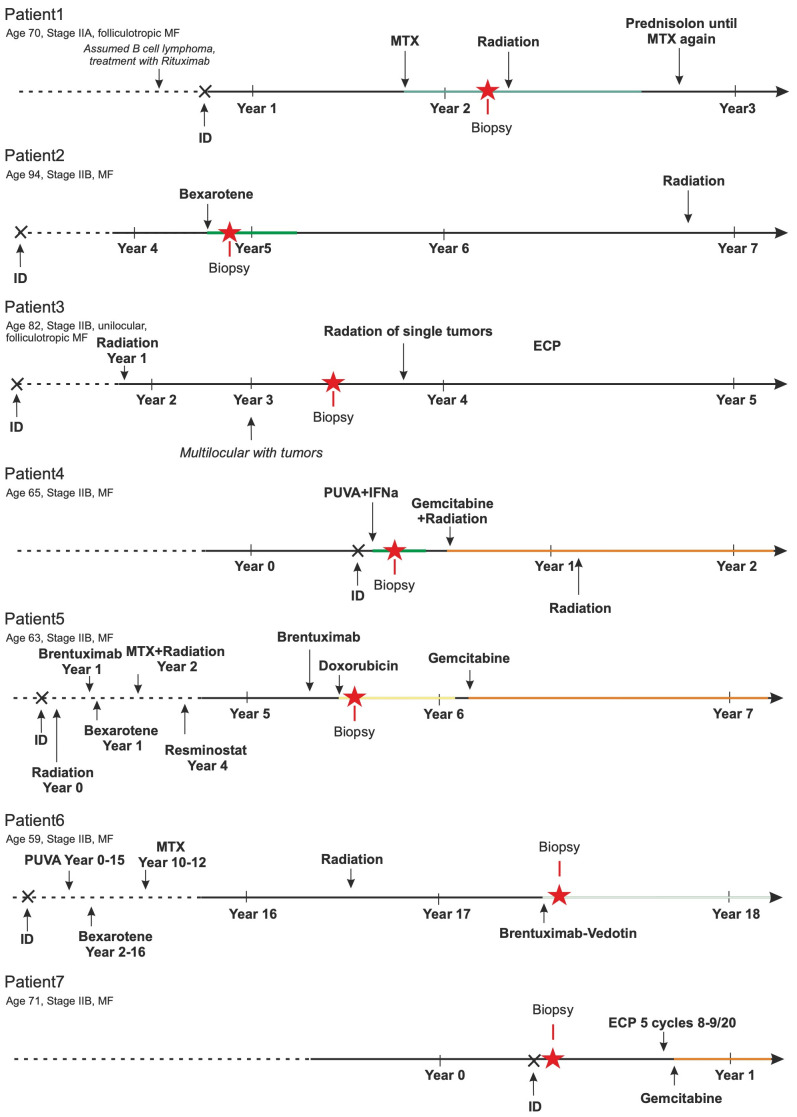
Patients’ history. The MF-specific history of these 7 patients is sketched. Duration of disease ranged from less than 1 to 17 years. Multiple different therapies (bexarotene, brentuximab-vedotin, doxorubicin, extracorporeal photopheresis (ECP), gemcitabin, interferon alpha, methotrexate (MTX), psoralen plus UV-A, resminostat, and/or ionizing radiation) were applied. The number of these treatments before biopsy collection ranged from treatment naïve to seven lines of therapy.

After processing fresh, native tissues obtained from cutaneous MF plaques or tumor lesions of 12 patients into single-cell suspensions, 9 of these suspensions passed the predefined quality control criteria. These criteria required the suspensions to contain more than 90% viable cells, with at least 30% being CD3+ T cells. Two samples had to be excluded from further analysis as one patient also suffered from B-CLL and in the other, the initial diagnosis of CTCL was revised to a fixed toxic drug eruption (**Supplementary Figure 1A**). Histomorphologically, there is a diffuse and dense infiltration of atypical lymphocytes in the dermis. These lymphocytes have irregular, hyperchromatic, cerebriform nuclei and prominent nucleoli, often extending into the subcutaneous fat. In some instances, papillary dermal fibrosis is evident. Despite the plaque/tumor stage, most cases exhibit some degree of epidermotropism, including the formation of Pautrier’s micro-abscesses (**Supplementary Figure 1B**). **Figure 1** depicts the individual disease course, including the therapies used, for each of these 7 patients. As commonly observed for cohorts of MF patients, they had widely varying disease histories, e.g., disease duration varied from less than one to 17 years and the number and type of therapies administered.

### 3.2 Single-cell transcriptomics of malignant and reactive T cells

**Figure 2:**
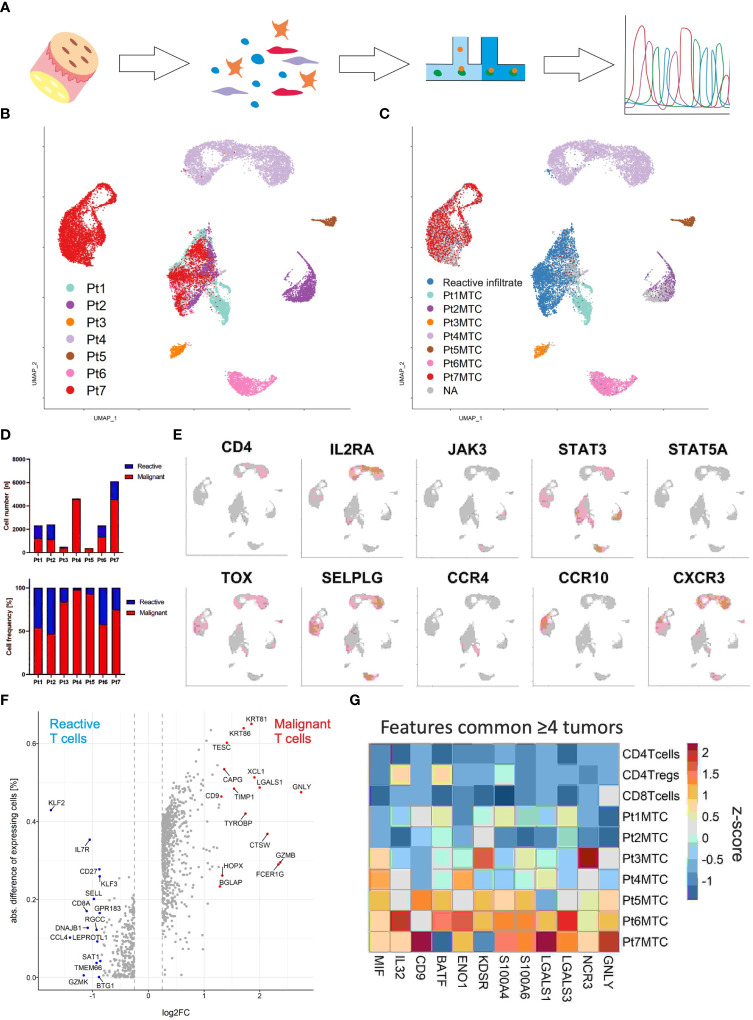
Single-cell transcriptomics of T cells isolated from MF lesions. **(A)** Workflow from sample preparation to sequencing. **(B)** UMAP for scRNA expression of 18,630 T cells isolated from cutaneous lesions of 7 patients diagnosed with MF. Cells were clustered using Seurat and annotated according to their sample origin. **(C)** TCR sequencing based clonotype information was applied to the UMAP. The most frequent TCR clonotype within a sample is assumed to be the respective malignant T-cell clone (MTC) and color coded accordingly; the polyclonal reactive infiltrate is depicted in blue. **(D)** Absolute (upper panel) and relative (lower panel) MTC to the reactive infiltrate ratios per sample. **(E)** Feature plots showing the normalized mRNA expression level of genes encoding commonly reported CTCL markers by incremental red shading. **(F)** Volcano plot depicting differentially expressed genes between malignant and reactive T cells for all samples combined. Differentially expressed genes were calculated by Wilcoxon rank sum test (logFC > |0.25|, adjusted P value < 0.05). **(G)** Differentially expressed genes between malignant the T-cell population and their respective reactive infiltrate are depicted in a heat map as averaged and subsequently scaled expression if differentially expressed in four or more tumors.

Malignant and reactive T cells freshly isolated from cutaneous MF lesions were scrutinized by combined scRNA and scTCRseq (**Figure 2A**). To ensure an unbiased analysis, we did not enrich any cells prior to single-cell analysis. Instead, we aimed to ensure that samples contained a sufficient number of malignant MF cells as well as reactive T cells. We proceeded with scRNAseq only if the single-cell suspension contained at least 90% viable cells, with at least 30% being CD3+ T cells. A lower proportion of T cells would result in an insufficient number of sequenced MF and reactive T cells, compromising the reliability of data on tumor cell heterogeneity and the T-cell microenvironment. Cells were annotated according to cell type specific marker gene expression using the Panglao Database as reference (**Supplementary Figure 2**). From a total of 36,722 cells of all patients, 18,630 were identified as T cells based on their expression of CD3 genes. Initial analyses were focusing on T cells. Clustering of the cells and the visualization using uniform manifold approximation and projection (UMAP) revealed one large cluster mixed with cells from all patients and seven distinct clusters with cells originating from only one patient each (**Figure 2B**). Consideration of the TCR sequencing data demonstrated that the mixed large cluster consisted of polyclonal T cells, whereas the individual clusters derived from each patient consisted of only one clonal T-cell population each (**Figure 2C**). Interestingly, the cells in the distinct cluster of patient 7 expressed a clonal, but non-productive TCR (**Supplementary Figure 3**). The proportion of the malignant MF cells was high in all patients, but varied substantially, ranging from 47% to 98% (**Figure 2D**).

Given the clear differentiation between malignant T-cell clones and the corresponding reactive T-cell infiltrate in every patient, we could then proceed to compare mRNA expression of established CTCL marker genes between these two distinct cell populations within cutaneous MF lesions (**Figure 2E**) (14). Specifically, we examined the expression of *CD4, IL2RA*, *JAK3*, *STAT3*, *STAT5A*, *TOX*, *SELPLG*, *CCR4*, *CCR10* and *CXCR3*, and found that only *STAT3* mRNA was expressed in levels that could be reliably detected by scRNAseq in the malignant T-cell clones of all MF samples. Nevertheless, the transcription factor *TOX* and the chemokine receptor *CXCR3* were detected in the malignant clones of 6 of 7 patients (**Figure 2E**); genes associated with skin homing or retention, *SELPLG* (coding for CD162, aka CLA) and *CCR10* were also expressed in most lesions, but to variable degrees. To determine whether the expression pattern of other genes may be better suited to distinguish malignant from reactive T cells by scRNAseq, we assessed differential gene expression in the pooled data from all patients (**Figure 2F**). Surprisingly, the most discriminative gene expression signature for malignant T cells was related to the mechanism or regulation of T-cell cytolytic activity such as *GZMB* (granzyme B), *CTSW* (cathepsin W, lymphopain) and *GNLY* (granulysin) (**Figure 2F**). Other genes with a stronger mRNA expression in MF cells were *LGALS1* (galectin-1), which suppresses Th1 immune responses (15), *CD9*, a tetraspanin involved in T-cell activation, migration and exosome biogenesis (16), and the keratins *KRT81* and *KRT86*, both mediating cell migration and tissue invasion (**Figure 2F**) (17). Genes with a higher mRNA expression in the benign T cells infiltrating MF lesions were members of the Krüppel-like family of transcription factors *KLF2* and *KLF3*, as well as genes associated with T-cell memory function, including *IL7R*, *CD27* and *SELL* (CD62L).

To overcome a possible bias in differential gene expression analysis by using the compiled data set of all patients (e.g., due to the variable number of malignant T cells), we next performed the analysis between reactive and malignant T cells of each patient individually. Twelve genes were differentially expressed and upregulated in the malignant cells present in four or more lesions (**Figure 2G**). Among these were pro-inflammatory genes associated with T-cell activation and differentiation, such as *BATF* (18), *IL32* (19), *MIF* (20), *CD9* (21), and genes involved in T cell metabolism *ENO1* (enolase 1) (22) and *KDSR* (3-ketodihydrosphingosine reductase) (23). The AP-1 family transcription factor BATF specifically mediates the differentiation of T-helper 17 cells (Th17). IL-32, MIF and CD9 are involved in the polarization of myeloid cells, with which the malignant cells may interact via NKp30 (encoded by *NCR3*) to stimulate their own growth (24). Interestingly, both glycolytic enzymes *ENO1* and *KDSR* have been shown to mediate the metabolic adaptation to hypoxic stress - a likely feature of the cutaneous microenvironment (22). The two S100 genes *S100A4* and *S100A6*, which are involved in many cellular processes including inflammation and cancer progression, were also highly expressed in the malignant T cells (25, 26). Finally, the strong expression of mRNA encoding galectin-1 and 3 in malignant T cells suggests the secretion of these proteins; both have been shown to suppress anti-tumor responses, favor Th2 inflammation and polarize the epidermal environment to a pro-tumorigenic state (27).

### 3.3 Inter- and intra-patient heterogeneity of MF cells

**Figure 3:**
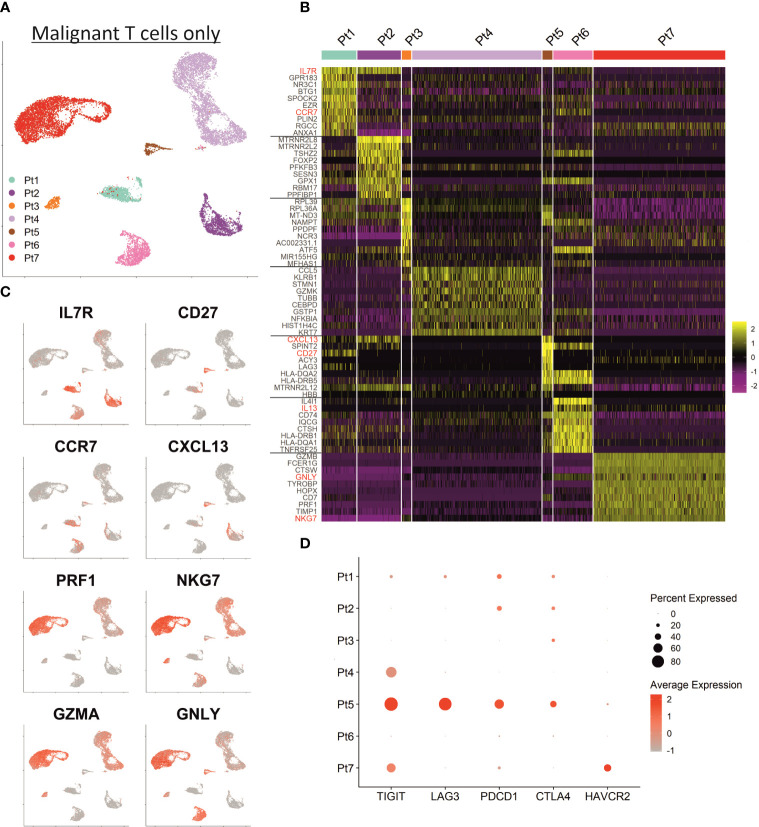
Inter-patient heterogeneity of MF cells. **(A)** UMAP depicting only the malignant T cells of all patients annotated by patient. **(B)** Heat map showing the expression of the ten top differentially expressed genes between MF cells of individual patients. Genes encoding established MF-associated markers are highlighted in red. Scaled expression values are color-coded with high expression in yellow and low expression in purple. **(C)** Feature plots depicting the normalized expression of indicated genes by incremental red shading. **(D)** Dot plot for expression of genes associated with T cell exhaustion in MF cells from each patient. Size of the dots reflects the percentage of cells expressing the respective gene and color intensity the relative expression intensity.

Shared nearest neighbor clustering separates malignant T cells from individual patients, indicating substantial transcriptional differences between patients’ malignancies. To scrutinize differences and commonalities, we focused exclusively on the malignant T cells: the two-dimensional presentation of mRNA expression profiles by UMAP already revealed clear differences between the malignant T cells of individual patients (**Figure 3A**). Indeed, most of the genes mentioned above were expressed markedly distinct expression levels among the different patients. **Figure 3B** depicts a heat map of the top 10 differentially upregulated genes in MF cells between patients demonstrating a unique mRNA expression profile for each individual patient. In detail, MF cells of patient 1, 2, 5 and 6 possess a transcriptomic phenotype characteristic for memory/naïve T cells characterized by expression of *IL7R*, *CCR7* and *CD27* (**Figure 3C**). MF cells of patient 1, 2 and 5 additionally express mRNA coding for the chemoattractant *CXCL13*, which has been described to enhance the migratory capacity of CTCL cells (28). Patient 6’s MF cells express the cytokines *IL4* and *IL13*, both associated with a Th2 phenotype (29). In contrast, malignant T cells of patients 3, 4 and 7 express genes associated with the regulation or execution of cytolytic activity such as *GZMA*, *GNLY* and *NKG7* (**Figure 3C**); in patient 7, MF cells also strongly express *GZMB* and *PRF1* mRNA. The high expression of glycolysis-related genes in patient 3’s tumor cells is likely caused by expression of high levels of *miR-155*, a microRNA promoting malignant T-cell proliferation (30). With respect to T-cell exhaustion markers, such as *PDCD1* (encoding PD-1), *CTLA4*, *HAVCR2* (encoding TIM-3), *LAG3*, *TIGIT* and *CD160*, which have been reported to be highly expressed in CTCL cells, we also observed a marked interpatient heterogeneity (**Figure 3D**) (31). For example, expression of *TIGIT*, polarizing toward Th2 immune responses, was detectable in only half of the patients (32). For *LAG3*, *PDCD1* and *CTLA4*, only patient 5 had a high number of expressing cells in the cutaneous MF lesion, whereas *HAVCR2* was only expressed in cells from patient 7 (**Figure 3D**).

**Figure 4:**
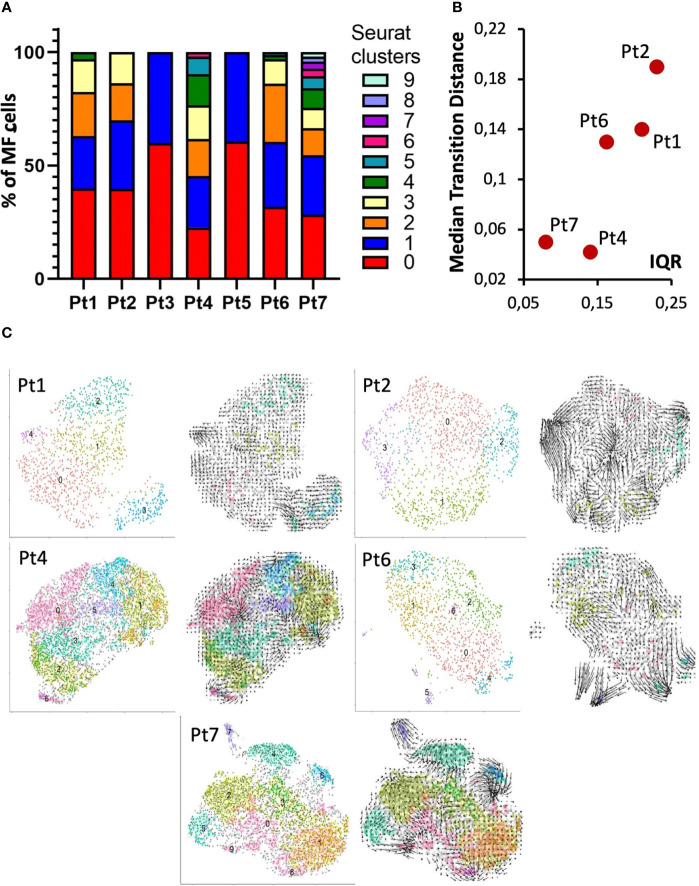
Intra-patient heterogeneity and transcriptional dynamics of MF cells. **(A)** Bar graph of transcriptomic diversity of the malignant T-cell population based on Seurat’s Louvain clustering for each patient. **(B)** Each panel depicts the indicated patient’s MF cells in a UMAP annotated according to Louvain clustering (left) or with arrows reflecting RNA velocities. **(C)** Dot plot to visualize the quantification of combined RNA velocities for the individual patients. The median transition distance reflects the magnitude of transcriptional changes (i.e., the velocity arrow length in PCA space) and the inner quartile range (IQR) the variance between the cells within a sample.

The heterogeneity of MF cells within a patient is also reflected by the number of clusters identified by shared-nearest-neighbor clustering (**Figure 4A**) (8). However, it should be noted that the number of identified clusters also depends on the number of sequenced cells, so for samples with a low number of sequenced MF cells, e.g., patient 3 and 5, this analysis should be interpreted with caution. These patients were therefore not included in the correlation of tumor heterogeneity with RNA expression dynamics. The latter was determined using velocyto (13), which allows to infer the direction and extent of transcriptional changes from the ratio of unspliced to spliced RNA. To quantify these transcriptional changes, we calculated the median transition distance in the PC space, indicating the magnitude of transcriptional change, and the inner quartile range (IQR), indicating the variance of transcriptional changes between all cells within a sample. This analysis again revealed large differences in the dynamics of mRNA processing between patients, thus providing additional evidence toward the heterogeneity within MF cells (**Figure 4B**). For depiction of cell transitions, we projected RNA velocities on the UMAP and used vector fields to combine individual cell velocities into neighboring group velocities (**Figure 4C**). These visualizations demonstrate that, for example, the highly diverse malignant T-cell population in patient 4 appears to be transcriptionally rather static, whereas the less diverse MF cell population in patient 2 shows considerable transcriptional dynamics.

**Figure 5:**
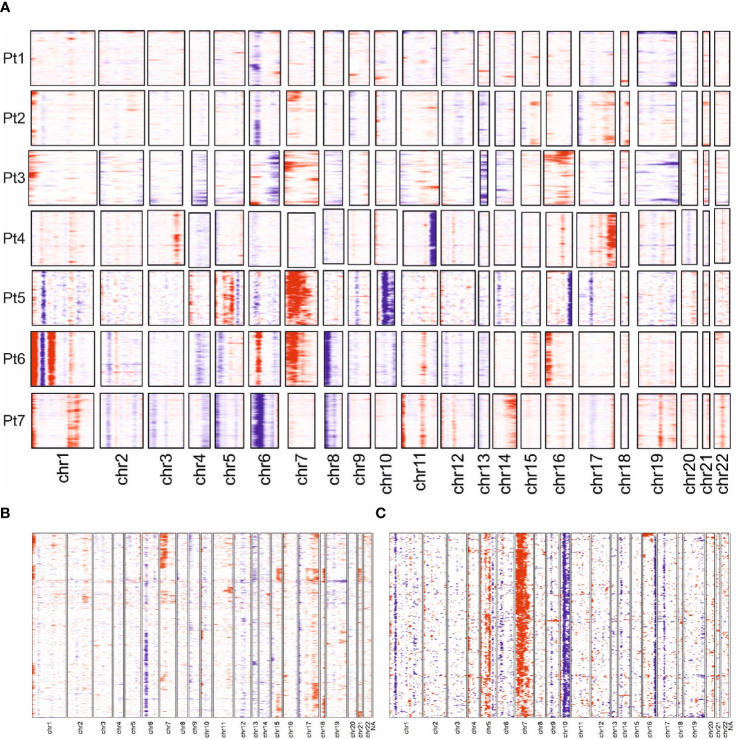
Copy number variation profiles in MF cells. **(A)** Overview presentation of scRNAseq data-derived CNV profiles of MF cells from all 7 patients. Regions with predicted copy number gains are indicated in red, losses in blue. **(B, C)** More detailed presentation of inferred CNV for patient 2 and 5.

The observed transcriptomic differences between and within individual patients may be due to the previously reported genetic heterogeneity observed in late-stage MF; indeed, only few common driver genes were identified (3, 8, 33). To test this notion, we derived copy number variations (CNVs) from the single-cell transcriptome data using the inferCNV algorithm. Sequencing data from the benign reactive T cells served as the normal reference. This analysis demonstrated that each malignant T-cell clone possessed an individual CNV pattern, which confirms the previous reports (**Figure 5A**). Of note, we observed more genetic changes in patients whose long-standing MF was treated with multiple therapies, e.g., patients 5 and 6; this suggests that the duration of the disease and the number of therapies, especially ionizing radiation, may result in more genetic changes (**Figure 1**). Amplifications were frequently found on chromosome 1p (*ENO1*, *TNFRSF1B*; n=5); deletions on chromosome 6 (*MHCI* and *MHCII*, n=4). However, the characteristic deletions of chromosomes 10q (*PTEN*, *FAS*, *NFKB2*) and 17p (*TP53*) reported for leukemic MF and SS were only observed in patient 5 and 2, respectively. The observed transcriptional heterogeneity of MF cells within individual patients was also reflected to some extent in the inferred CNV signature, as shown by the example of patients 2 and 5 (**Figures 5B, C**).

## 4 Discussion

Until recently, reliable differentiation between malignant and reactive T cells has been a major challenge in the comprehensive examination of MF cells *in situ* and *ex vivo*. Combined scRNA and scTCRseq solves this problem and has also been successfully used to characterize CTCL cells, but so far mostly from blood of patients with leukemic CTCL, *i.e.*, Sézary Syndrome (4, 8). Here, we report that we were able to isolate sufficient numbers of viable T cells from skin lesions of seven MF patients to establish the single-cell RNA expression profile and TCR sequences of 13,592 malignant and 5,038 reactive T cells. Clonal rearrangement of TCR unambiguously identified malignant T cells. Notably, in one of the patients, we identified an expanded T-cell clone with a nonproductive TCR VDJ rearrangement; its malignant transformation may have thus occurred early in lymphocyte development (34). The identity of the malignant T cells was further confirmed by the presence of specific CNVs inferred from scRNAseq data. This analysis demonstrated that each malignant T-cell clone had an individual CNV pattern, in line with previous reports of the genetic heterogeneity of CTCL (3, 8, 33). For some patients inferred CNV also demonstrated marked differences among the malignant T cells present in the individual lesions, which correlated with individual transcriptional signatures, suggesting the involvement of different oncogenic drivers and signaling pathways. Genomic instability has been proposed to be a feature of CTCL based on the aberrant functions of genes involved in genome maintenance and DNA repair (35). In this context it should be noted that in our series the extent of genetic alterations increases with the duration of the disease and the therapies applied, especially ionizing radiation.

It is important to note that in our single cell transcriptomic study genes encoding classic MF markers were not always detected in all malignant T cells. This observation is consistent with the fact that mRNA expression does not correlate linearly with protein expression (36). However, we were able to identify gene expression signatures highly discriminative between malignant and reactive T cells: keratins (*KRT81*, *KRT86*), S100 genes (*S100A4*, *S100A6*) and galectins (*LGALS1*, *LGALS3*) were all overexpressed in CTCL cells. *KRT81* was originally described to be involved in hair follicle formation and growth, but was since then also linked to cancers, including non-Hodgkin lymphoma and melanoma (37). Its paralog *KRT86* has been associated with T-cell exhaustion (38). *S100A4* and *S100A6*, both of which have pro-inflammatory and pro-tumorigenic properties (25, 26) were expressed in malignant but not in reactive T cells. Galectin-1 and 3 are also highly expressed in MF cells; galectins promote lymphocyte survival and have been associated the Th2 differentiation (39). Our observation is in line with previous reports that demonstrated higher expression of S100 family and galectin genes, specifically within the malignant cells (3, 40). These studies have revealed the presence of distinct gene expression patterns within malignant T cell subclones, but their functional relevance in CTCL remains to be investigated (3, 33). On the other hand, while genes coding for skin homing molecules such as *CCR4* and *SELPLG* (CLA) were expressed by almost all malignant T cells, they were also detected in T cells of the reactive infiltrate.

The heterogeneity of MF cells is also reflected in their functional characteristics. It is generally accepted that malignant T cells in SS resemble central memory T cells, whereas those in MF are more similar to skin resident effector memory T cells (41). The single cell transcriptome of MF cells isolated from cutaneous lesions, however, suggests additional phenotypes: in 4 patients MF cells expressed genes associated with memory T cells such as *CD27*, *CCR7* and *IL7R*, whereas in 3 patients we observed gene signatures typical for cytotoxic T cells, including expression of *GZMA*, *NKG7* and *GNLY* (3). Although CD4^+^ cytotoxic T cells were once thought to be an *in vitro* artifact and even today the cytokine and transcription factor requirement for their differentiation mechanism remains largely elusive, CD4^+^ cytotoxic T cells have been identified *in vivo* and shown to play important roles in adaptive immunity (42). Differentiation into CD4^+^ cytotoxic T cells may be caused by chronic antigen stimulation and depends on the transcription factors T-bet and eomesodermin encoded by the *EOMES* gene possibly induced by high amounts of IL2 present in MF lesions (43). Still, this is an unexpected observation since differentiation into cytotoxic CD4^+^ T cells generally occurs under Th1-skewed conditions, which in MF are only present in very early stages (44). Indeed, disease progression is associated with an increase in Th2 markers including transcription factors (GATA-3) and Th2 cytokines such as IL4 and decreased expression of Th1 markers including T-bet, IFN-γ and IL12 (45). Since we observed expression of typical Th2 cytokines IL4 and IL13 as well as drivers of Th2 inflammation such as *TIGIT* (32), other mechanisms have to be operative to drive differentiation toward the cytotoxic CD4^+^ T cell phenotype. To this end, the expression of NK-cell receptors on malignant T cells of CTCL is well established (24). Expression of genes coding for NK-cell receptors including *NCR3* (NKp30) in the malignant T cells was present in five patients, which can be explained either by enhanced Myc signaling or high IL15 expression in the tumor microenvironment (46). Myc signaling has been shown to promote cytotoxic CD4+ T cell differentiation (47).

In conclusion, our comprehensive exploration through unbiased scRNA and scTCRseq of cutaneous MF lesions uncovers a striking landscape of high inter- and intra-patient heterogeneity among MF cells. What’s more, we have unveiled transcriptomic signatures derived from scRNAseq that serve as powerful discriminators between malignant and reactive T cells. Remarkably, the transcriptome expression patterns in certain patients hint at the potential for a shift toward a cytotoxic phenotype in MF cells, in addition to the conventional skin-resident effector memory T cell phenotype. Our study is constrained by the fact that the recruited, unselected MF patients who presented for consultation, had undergone diverse therapeutic interventions prior to tissue sampling. While obtaining longitudinal samples from similarly treated MF patients at different, defined time points would provide better insights into the disease’s inherent heterogeneity, this approach poses significant challenges, especially in a rare disease. Additionally, to isolate a sufficient number of malignant and reactive T cells from skin biopsies, patients had to have at least MF plaques, i.e. a time point when most patients already had received several lines of therapy. Consequently, our current approach relied on a representative collection of samples in a real-world setting, limiting our ability to control for treatment variability. Despite these constraints, our study provides insights into the heterogeneity of MF lesions, including the tumor microenvironment, in a real-world setting likely influenced by heterogeneous therapeutic histories. We acknowledge that future studies with more controlled treatment variables will add valuable insights. Moreover, while scRNA/TCRseq provides valuable insights into the transcriptomic landscape of MF, its application is largely restricted to more advanced lesions due to technical limitations and the need for sufficient cell numbers. However, the emergence of spatially resolved transcriptomics techniques offers a promising solution for the analysis of earlier, less cellular lesions. By leveraging the established transcriptomic signatures derived from scRNAseq studies of advanced MF as presented here, spatially resolved transcriptomics can be applied to early-stage lesions. This approach allows for the preservation of spatial context while providing high-resolution molecular information, potentially enabling more accurate differentiation between early MF and benign dermatoses. The combination of these complementary technologies promises to enhance our understanding of disease progression and improve early diagnostic capabilities in cutaneous T-cell lymphomas. Still, our findings not only underscore the imperative for tailoring individualized therapeutic strategies for MF patients but also lay a robust foundation for more profound mechanistic inquiries, with the promise of advancing treatment options for this condition.

## Data availability statement

The original contributions presented in the study are publicly available. This data can be found here: https://ega-archive.org/studies/EGAS50000000226.

## Ethics statement

The studies involving humans were approved by the ethics committee of the University Duisburg-Essen (18-8230-BO), which was conducted in accordance with the Declaration of Helsinki. The studies were conducted in accordance with the local legislation and institutional requirements. The participants provided their written informed consent to participate in this study.

## Author contributions

NS: Visualization, Writing – original draft, Writing – review & editing, Conceptualization, Data curation, Formal analysis, Investigation, Methodology. LP: Conceptualization, Data curation, Formal analysis, Investigation, Methodology, Software, Visualization, Writing – original draft, Writing – review & editing. KH: Formal analysis, Software, Visualization, Writing – review & editing. KL: Formal analysis, Software, Visualization, Writing – review & editing. TB: Formal analysis, Software, Writing – review & editing. LK: Methodology, Writing – review & editing. ML: Investigation, Methodology, Writing – review & editing, Validation. MY: Investigation, Software, Writing – review & editing. IS: Investigation, Methodology, Writing – review & editing. AS: Data curation, Resources, Writing – review & editing. FF: Formal analysis, Software, Supervision, Writing – review & editing. JK: Supervision, Validation, Writing – review & editing. SU: Data curation, Investigation, Resources, Validation, Writing – review & editing. EL: Data curation, Resources, Validation, Writing – review & editing. TG: Data curation, Resources, Validation, Writing – review & editing. NØ: Formal analysis, Supervision, Writing – original draft, Writing – review & editing, Conceptualization. JB: Formal analysis, Funding acquisition, Investigation, Methodology, Resources, Supervision, Writing – original draft, Writing – review & editing, Conceptualization.
