# Supplementary figures and images for: Single-cell RNA and T-cell receptor sequencing unveil mycosis fungoides heterogeneity and a possible gene signature
Source: Front Oncol. 2024 Aug 7;14:1408614. doi: 10.3389/fonc.2024.1408614 (PMC11337020; doi:10.3389/fonc.2024.1408614)

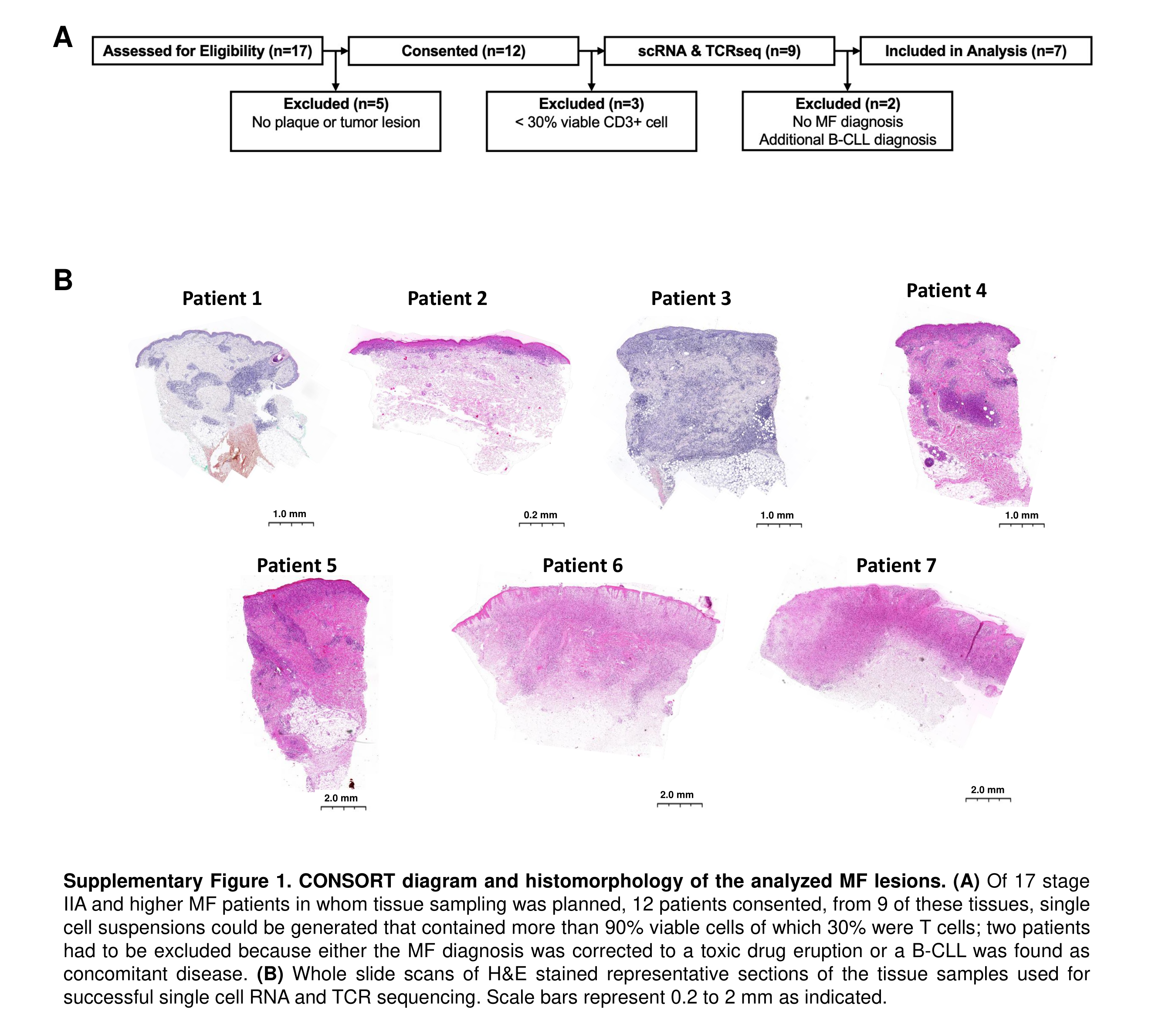

Supplement: Supplementary file 1 [file Image_1.tif]

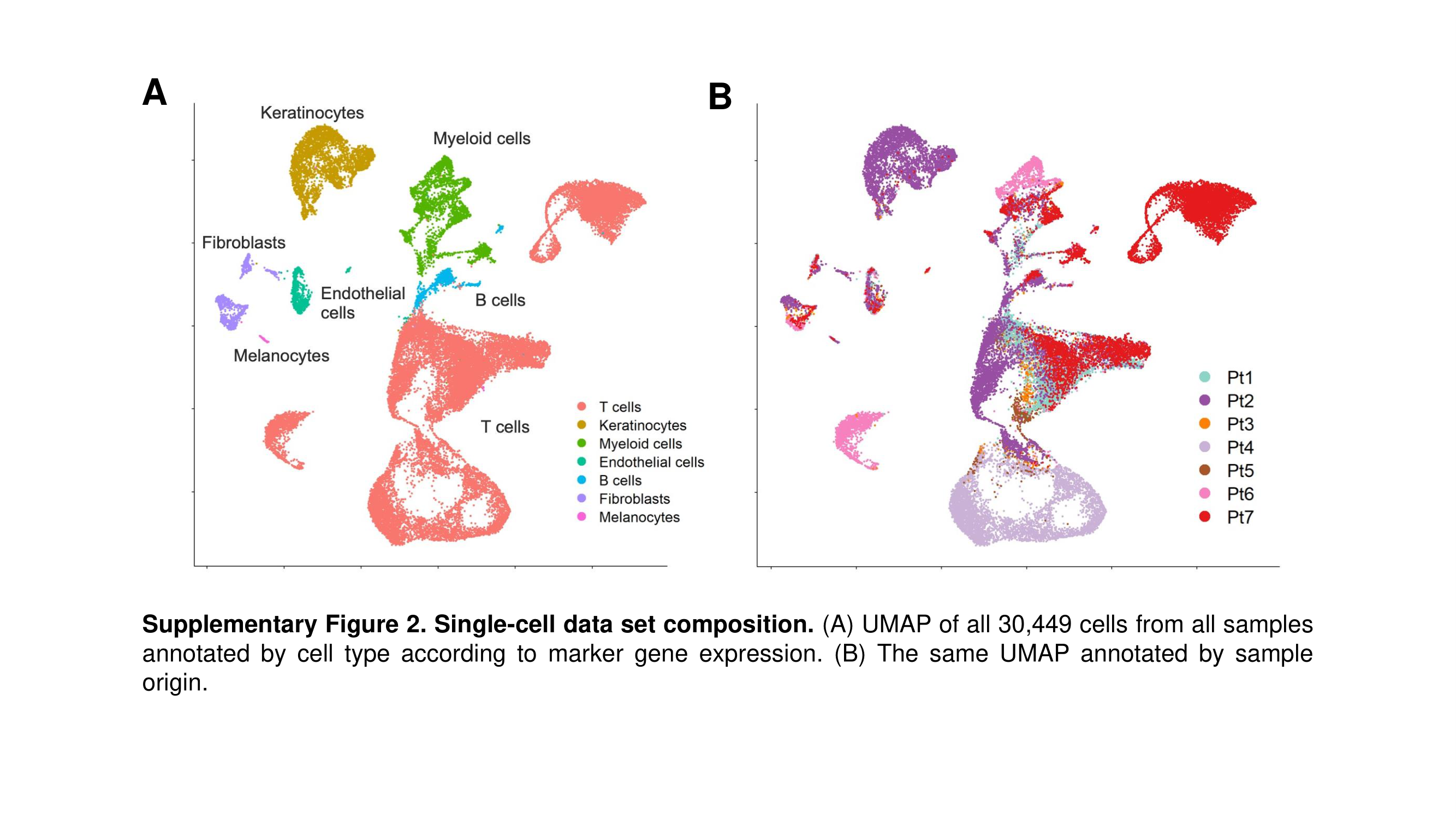

Supplement: Supplementary file 2 [file Image_2.tif]

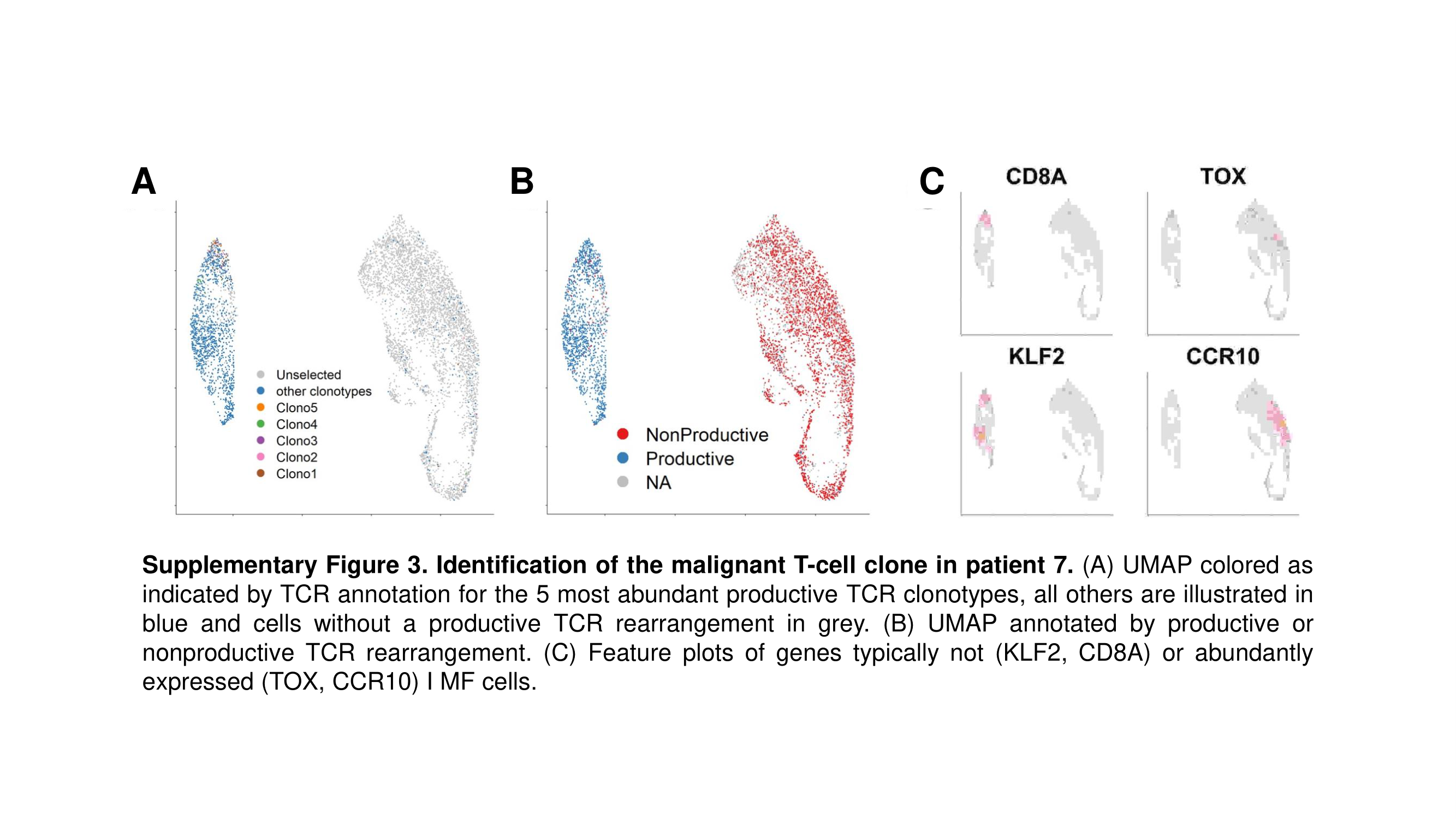

Supplement: Supplementary file 3 [file Image_3.tif]
